# Translations from Foreign Exchanges

**Published:** 1883-08

**Authors:** 


### Translations from Foreign Exchanges.

By O. Stroinski, M.D., Chicago.

Cotoine.

Cotoine, recently detected in the cota bark by Sabot, when given to healthy persons in doses of ½ gram., increases the appetite without producing constipation. It is easily dissolved in the stomach, but slowly in the alkaline fluid of the intestines. It will be found in the urine, giving a yellow-collored deposit by alkalies, a blood-red by nitric acid, and a yellowish-brown by sulphuric acid. Albertoni (*Annali. Universali Di. Med. e Chir.*) reported his results with cotoine in a series of 100 cases, the most of his patients being insane or very emacated persons. To acquire correct results he made strict rules, regulating the nourishment, temperature, etc. He comes to the following conclusions: Cotoine is indicated in diarrhœa, complicating the various forms of insanity. It is especially the neuro paralytic diarrhœas which can be artifically produced by depriving the intestinal tube of its nerves. It is proved that in the diarrhœa of the insane, the lessened faculty of resorption is the principal point, and also in chronic intestinal catarrh; in which cases cotoine is a good remedy. In diarrhœa of cachectic or marasmic persons, in malaria, and in the diarrhœa of nurslings, cotoine is indicated, as it has also given good results in pellagra. It is given in doses of 15 to 20 ctgrm., in different cases, every four hours. The best form is the powder, but it must be the genuine article. Albertoni thinks that it has a specific influence on the epithelial coat of the intestines, modifying the physiological action of the latter. He compares the whole intestinal tract to a gland whose epithelium attracts the digested matter, and conducts it into the blood. If the physiological action of the epithelium is lessened, as in intestinal catarrh, etc., the epithelium does not only absorb nothing, but it admits a filtration of substances from the blood. Cotoine acts directly on the epithelium, and it produces thus very good effects.—*Med. Chir. Centralblatt.*

JEQUIRITY-OPHTHALMIA. A NEW INFECTIOUS DISEASE.

L. De Wecker first called the attention of ophthalmologists to the effects of a decoction of the seed of abrus pecatorius on the conjunctiva of the eye. The fact that in cases of long existing chronic inflammation, an acute attack removes the old exudation by resorption of the latter, is well known to the medical world, but there has never been a better proof of this fact than the effects of jequirity on the conjunctiva. Since the inoculation of acute blenorrhagic conjunctivitis in cases of trachoma, etc., which is a very prompt but at the same time dangerous proceeding, several remedies have been used to provoke an acute inflammation on the conjunctiva, but without success. Jequirity has been known in Brazil for a long time, and the reports of Brazilian physicians show the dangerous character of this remedy, if it is not well regulated by experts. Of the seeds, reduced to a fine powder, about 3 grammes are macerated in 500 l.l. of cold water for 24 hours, then the same quantity of hot water is added, and the whole filtered. The fluid is applied with a brush to the lids three times every second day, until inflammation is produced. By close observation, it has been determined that this inflammation shows a different character from all the other typical inflammations of the conjunctiva—that it is, in fact, an inflammation *sui generis*. Dr. Tattler, in making repeated experiments and microscopical examinations, has proved that the inflammation is caused by micro-organisms contained in the seeds of jequirity. This experimental study has been confirmed by the contagious character of the inflammation. All persons whose eyes came in contact with the instruments used in the experiments, or with persons treated with jequirity, have been likewise attacked by conjunctival inflammation.—*Wiener Med. Wochenschrift.*

CONVALLARIA.

Linné says in his Flora Lapponica : "ne inventionen nimiam introducamus levi mutatione *convallariam* decimus aliorum *Lilium convalliam*" (the lily of the valley). This plant was known in the dark ages and it is mentioned in the Bible. Long before Christ the flower was much praised among the then savage Germans and it has at this day many high-sounding German names. After the conversion of the Germans to Christianity, the flower got the name of the mother of Christ (Mary's flower), an indication of the respect in which the flower was at that time held. In the year 1029 A. D., Bishop Falbertius said: The feast of the birth of the holy Maria should be celebrated especially by the virgins, for on that day is annually born (is the birth) the lilium convallium. And Saint George, the noble Hungarian, is painted in full armor, having conquered the dragon with the lilium convallium. In those dark ages the convallaria was regarded as a regular panacea and the decoction of it was called aqua aurea (golden water). In the castle of Wolfartsweiler there is secreted a large treasure, to recover which there appears every seventh year a lady carrying a bunch of convallaria. Of all the grandeur of this highly praised plant there remained in the last century only its value as a snuff. But this day of railroads and telephones has brought again to light the ancient glory of convallaria, and the pens of many professors are busy in describing its wonderful effects. Do you wish to know who you are, then look about you on the faces of your friends. The convallaria belongs to the same class as atreptopus, paris, smilax and rascus, whose medical effects are null. Rest in peace dear convallaria.—*Med. Chir. Central-Blatt.*

COMPRESSION OF THE OVARY IN RELATION TO HYSTERICAL ATTACKS.

The experiments of Charcot at Paris, with this object in view, have not been regarded as very important and truthful, but the following case corroborates his views : The patient, a girl twenty-three years of age, was the daughter of nervous and uræmic parents. She menstruated when fourteen years of age, with severe pains, which increased monthly, and in her seventeenth year she had hystero-epileptic attacks. After a certain time the attacks disappeared, but she passed now through all the symptoms characteristic of hysterical persons. The uterus was enlarged and strongly anteflexed, and there was in the anterior wall a fibroid tumor of the size of a walnut, causing complete retention of the urine. The anteflexion was treated by inserting an intra-uterine stem pessary of glass. One day the patient fell from a chair and the intrauterine pessary was broken to hundreds of pieces. Two large pieces were removed, but it was impossible to extract the many small fragments of glass which were imbedded in the mucosa. From this time on, the patient had severe nervous attacks. She did not recognize her parents, and she thought the physician to be one of the servants, writing at the same time to him and complaining about his neglecting her. At other times she left her bed, and she played the most difficult compositions on the piano. In one of these attacks there was made a bimanual compression over the left ovary, and the patient suddenly became conscious, recognizing everybody around her, and speaking in the usual polite manner, while before she had been very abusive.—*Memorabilien*,

PARAMYO-KLONUS MULTIPLEX.

The patient, 10 years of age, is of healthy parents. When 4 years of age, for years after measles, he suffered from dyspeptic troubles. Two years ago he had contraction of the right arm, especially on the flexors and extensors of the carpus, and the patient was not able to use this hand in writing, etc. Later, the left arm was attacked. The present status is as follows : No irregularity on the part of the cerebral nerves, skin intact, no symptoms of muscular atrophy or hypertrophy. No peculiar sensibility, a pressure on the skull or vertebral column and no sign of ataxia. In observing the undressed boy there is noticed a peculiar muscular movement of the upper extremities. The supinator longus and the biceps present a perpetual change of contraction and relaxation of these muscles. In the supinator there are counted 40 contractions in 30 seconds. The pectoralis major is also slightly contracted on the left side. On the lower extremities there is the same peculiar change. The muscles affected in these parts are the vastus internus, gracialis, semitendinosus and semimembranosus. In the latter are counted 36 contractions in 15 seconds. By active movement the normal state appears immediately. Cold air or pressure on the muscles increased the contraction considerably. The muscles acted normally when electricity was applied. The patient was treated with the galvanic current and the success was apparent after including the ganglion supremum in the current and by the internal use of vaterianiate of zinc.—*Aerztl. Intel. Bl.*

THOMSEN'S DISEASE.

In the society of physicians at Berlin, Prof. Westphal presented two cases of this very rare disease, It is shown by tonic contractions of the muscles and the patients feel a tetanic stiffness in the extremities, increased by complicated movements. Passive motion is easily executed and there is no disturbance in sensation. This anomaly of motion is extended to muscles provided by the cerebral nerves and there is stiffness of the tongue, difficulty in opening the eyes, and muscular tension in masticating. The sphincters are free. The most prominent symptom in this disease is the greatly increased volume of all the muscles of the body, so that tbe patients look like athletes. But the muscular power not being increased, the patients are usually looked upon as lazy people, and they have as a consequence been badly treated in the German army. The tonic contractions are very slow and in tapping the biceps one will see the muscle contract to a very large bundle, but it will recede very slowly. The pathology and the therapy of this disease are entirely unknown, but the disease is apparently hereditary in character.—*Wiener Med. Wochenschr.*

ANGUILLULA STERCORATIS AND ANGUILLULA INTESTINALIS.

Cuckart, at Leipsic, detected anguillula, which had been first described as causing diarrhœa in Cochinchina, in the fœces of a man who had lived for a long time in Mexico and Atchin. He transferred the young worms to a breeding apparatus, and after thirty hours there were developed the full-grown worms, provided with a complete genital apparatus. The animals copulate outside the intestines, and the females produce ova and embryos, which readily escape, the young worms measuring from 02 to 05 mm. After throwing off the skin, they have the characteristic form of rhabditis, and they take the form of strangylids, distinguished from the former by the tail and æsophagus. But this generation does not develop, and it is most probable that they have to be introduced into the intestines to reproduce the characteristic form of rhabditis. It is further probable that this generation will not be developed to anguillula stercoratis, but to anguillula intestinalis, and that the former has to be stricken from the class of poro-parasites inhabiting the human body.—*St. Petersburg Med. Wochenochraft.*

ENCEPHALIC HÆMORRHAGE FROM LESIONS OF THE MEDULLA OBLONGATA.

M. Brown-Séquard reported in the Biological Society of Paris that he had seen lately several cases of hæmorrhages into the lung, liver and heart, caused by traumatism of the brain in consequence of a shock to the posterior part of the cranium. He has seen the great trunks of the arteries and veins of the lung to contract rapidly, and to drive the blood into the capillaries so as to burst these vessels. The reverse will be seen by section of the inferior part of the medulla clavicalis in the pigeon producing instantly hæmorrhage at the end of the bulbar portion. The pathogeny of these accidents is under the influence of the vaso-motor nerves. In the same manner must be explained cerebral hæmorrhage appearing suddenly in several points, and which it is difficult to attribute to a simultaneous rupture of a great number of miliary aneurisms.—*Le Progrio Médical.*

MALIGNANT TUMORS OF THE THYROID GLAND.

The mortality of operations upon malignant tumors of the thyroid gland is very large. In all cases where there is the least suspicion that the vessels of the throat or the trachea or œsophagus are diseased, all these parts have to be extirpated. Of 34 patients operated on, 22 died right after operation, and there is but a single case known where there was no relapse after 16 months. The diagnosis of malignant growths in the thyroid gland is very difficult, and it is often only at the operation that the diagnosis can be made correctly. The sarcoma, as well as the carcinoma and the adenoma, are found in the thyroid gland, and there are very often mixed growths. That excision of so important parts as the larynx is often followed by death, is not surprising.—*CorrespondenzBlatt f. Schweizer Aerzte.*

### URETHRO-RECTAL FISTULA, WITH NARROWING OF THE URETHRA AND INTERSTITIAL NEPHRITIS.

The patient, twenty-nine years of age, had suffered three times from blenorrhagia and narrowing of the urethra. He had difficulty in passing urine, with cystitis and orchitis. Afterward, he noticed that the urine passed through the rectal orifice, as well as through the urethra, and once he could urinate through the anus for ten hours. To pass a sound into the rectal fistula was impossible. Poultices on the abdomen and warm baths accelerated the flow of urine through the urethra. All at once, there was complete retention of urine, and shortly afterward he died. At the autopsy, there was found a fistula in the rectum, 3 cm. from the anus, which communicated with the bladder. The urethra had three narrowed points; one in the spongy portion of the canal, and two others in the membranous portion. In the bladder were profound ulcerations. Both kidneys were atrophied, and the intestines black with congestions.—*France Médicale.*

### COLD ABSCESS OF THE TONGUE

The patient has a round spheroid tumor, of the size of a nut, on the right side of the tongue. The tumor commences 2 inches from the point of the tongue, is not discolored, and seems to adhere to the mucosa. Being absolutely painless it is easy to palpate the tumor and even to knead it. There is no impression left upon or by the tests and there is no trouble either in mastication or in deglutition. The tumor was first noticed 4 months ago and it grew rapidly for the first month, but since that time it has been stationary. It was thought to be a hydatid cyst, but puncture revealed a sack filled with fetid pus. The sack was excised and the wound closed by 4 sutures healing per primam. —*France Mèdicale.*

THE CAUSE OF ICTERUS NEONATORUM.

Birch-Hirshfeld, after having examined 600 corpses of newborn children, attributes the symptom to hepatic origin. The immediate cause is œdema in the region adjacent to the vena porta, and œdema in Glisson's capsule, producing compression of the secondary ducts of the gall-bladder. This theory is supported by Hofmeister's researches, who found, generally, biliary acids in the fluid contained in the pericardium of newborn children who died from icterus. By experimental researches, he found that pressure in the region of the vena porta produces a thickening of the blood. He maintains that the infection takes its way by the umbilical vein, and not by the umbilical artery.—*Le Frazre's Médicale.*

ATROPINE IN EPILEPSY.

In the medical society at Vienna, Dr. Lesdesdorf presented a man, forty-five years of age, who had been treated with the bromide of potassium for a long time without success. He being of healthy parents, he never had been sick until he was attacked by a bull, who threw him several feet when he fell upon his occiput. From this time on he suffered from headache, became insane, and had from six to eight epileptic attacks daily. He took the bromide of potassium for two months with apparently no effect, but when he took some powders composed of 02 oxide of zinc and 0001 of atropine, the epileptic attacks did not return.—*Med. Chir. Centralblatt.*

BOTHRIOCEPHALUS.

M. Tenneson showed in the Academy a number of pieces of bothriocephalus resembling inverted intestines. They were expelled by a man who had lived for a long time on the borders of the lake of Geneva, and whose defecations contained a number of eggs of the bothriocephalus. The pieces had the gray median genital pores, and there could be no doubt that they belonged to bothriocephalus. Pelletierine was given with success.—*Le Praticien.*

The Treatment of Amenorrhœa in Morphine-Eaters.

The morphine should be suppressed first, but that alone is not sufficient to restore menstruation. It is necessary to give the patients daily a cold douche-bath, directed to the inferior part of the trunk and to the lower extremities. Before and after the douche they must walk. They should take pills composed of extr. belladonna 0.40, and sulphate of quinine 0.60 to .40 pills, five to ten daily. After a month's treatment, the uterine isthmus should be catheterized until menstruation sets in regularly.—*Rêvue Mêdicale.*

In the last surgical congress at Berlin, Dr. Lehoenborn reported a very interesting case of gastronomy. A girl, 15 years of age, suffered from incessant vomiting, and there was a hard tumor of the size of a fist below the liver. After dissection of the abdominal walls the tumor was found to be in the stomach, and adjoining the latter there was detected a black moveable body which proved to consist of small pieces of hair mixed with mucus. The girl confessed that she had bitten off from her cue the ends of the hair to get a clear voice. Recovery was rapid without any fever.

---

By A. Lagorio, m.d.

Imperforate Anus, with Normal Conformation of Rectum and Discharge of Meconium by a Retro-Scrotal Cutaneous Orifice.

Imperforations of the anus are divided in three classes: 1st, Simple imperforations; 2nd, Imperforations complicated with a partial or total absence of rectum; 3d, Imperforations complicated by a deviation of rectum, opening in other parts than the anal region, and forming an anus *contre nature.* The following case, that Dr. Ramonet reports in the *Archives de Gênérale de Médicine* (March, 1883,) does not belong to any of the above classifications; it constitutes a new variety, interesting and valuable to know, and was never described by any author. The patient is a new-born male child, three days old. On account of the imperforated anus he has not discharged but a very small quantity of meconium through an orifice of the size of the head of a pin situated behind the scrotal sac. Patient is quiet, is not agitated, the aspect of the face does not show any suffering; cries a little, but the cry has more expression of anger than pain. No meteorism, and no tenderness on pression; has no vomiting, temperature is normal, respiration and circulation regular.

These negative symptoms show a perfect tolerance on the part of the intestine; they singularly contrast the symptoms of an internal obstruction. The inspection of the anus shows an imperforation of this organ, with no signs of its location. The skin of the perineum is smooth, with no depressions, and without designating the median raphe. No fluctuation under the skin is detected, to show any accumulation of meconium. Pression is made on the abdomen to press down the meconium, but nothing of diagnostic importance is elicited. On the upper and posterior part of the scrotal sac, and on the median raphe of the scrotum, exist a small opening, through which the infant has expelled a little meconium. With no little difficulty a probe is introduced in this abnormal opening, and great precaution is used in pressing it inward. It passes horizontally *under the skin*, and reaches the spot where the anus should anatomically exist. This obstacle gave the exact place of the anus. The probe is withdrawn and found covered with meconium; the index finger is pressed in front and behind along the fistulous subcutaneous tract just discovered, and a little meconial substance is forced out of the small orifice. Diagnosis now became easy and the success of a surgical intervention was no longer to be doubted. The rectum had no deviation, was well formed, terminated at the proper place with an anus covered by the skin, and that, instead of opening directly out, formed a subcutaneous tail, represented by the fistulous tract, opening under the scrotum to give exit to the meconium. No time was to be lost and an operation was decided upon. The little patient was placed on the knees of an assistant, in a lithotomy position; a grooved sound was introduced into the scrotal orifice, and pushed cautiously along the fistulous tract until obstructed in the anal region. The skin was pressed against the point of the sound, and through it was made a longitudinal incision, from in front, backwards two centimeters long. This done, the sonnd was withdrawn. The finger, well oiled, was then introduced in the wound, and pushed toward the rectum. After a great effort to dilate the sphincter muscle energetically contracted, the finger passed in, and in the rectum. In operating the little finger is always to be used to dilate the sphincter, to conquer the spasm, and not withdrawn until the muscle is relaxed. The rectum now expelled a large quantity of meconium. A carbolized tampon was placed in the wound, and changed several times a day. The subcutaneous fistulous tract was left alone, and cleansed by pression and by injecting warm water, and was soon obliterated. The operation was a success.

A different operation could have been made. The fistulous tract could have been incised on a grooved sound down to the anal region, and then a circular piece of skin excised to make the anus. But the author did not think it best. Such an operation would have produced a long wound, which might have become ulcerated or gangrenous by coming in contact with the urine and fæces, and also severe danger of infiltrations of urine, and erysipelatous complication, so liable in the new-born. Lastly the following conclusions are reached :

1st. The case of imperforate anus just reported constitutes an interesting variety, to be added to the different kinds described up to date.

2d. The exit of fœcal matter by an abnormal cutaneous opening, situated in any part outside the anal region, is perfectly compatible with a normal conformation of the rectum, and does not absolutely implicate the deviation and aperture of this organ *contre nature*.

3d. In cases similar to the one reported, the surgeon will leave alone and not incise the fistulous tract ; but will use it as a conductor to make the perineal incision, which will form the anus.

HÆMETIC ORIGIN OF BRIGHT'S DISEASE.

The aim of the researches made by Prof. M. Semmola on Bright's Disease has been to demonstrate that in this disease, on account of the suppression of the cutaneous functions the albumen of the blood undergoes a chemico-molecular alteration, and not being either assimilated or burnt, absolutely must be eliminated from the body as a substance foreign to the organism. This elimination constitutes the fundamental fact of Bright's albuminuria. Prof. Semmole in his preceding works has studied the chemical reactions capable to characterize the different albumens of the organism as to differentiate them from that of Bright. From these preceding researches, and from those more recent, the author asserts that the albumen found in the urine of Bright's disease is undoubtedly different from that found in albuminuria of a cardiac or amyloic origin. An able expert in the analysis of urines will not mistake the color of the precipitate, and will know immediately the albumen of Bright's disease, which has a particular color, already described by the author (Paris, 1867). To demonstrate the reality of the alteration of the albuminoid substances of the blood, the author has studied the degree of diffusability of the albuminoid substances in the serum of the blood and not in the urine, and has demonstrated that in Bright's albuminuria the diffusion is more or less complete according to the gravity of the disease, while in the normal state, or in other albuminurias (not of Bright's disease) the diffusion is none or very little, without any relation to the quantity of albumen eliminated in the urine. This fact leaves no doubt of the initial existence of chemico-molecular alteration of the albuminoid substances of the blood in Bright's disease, chemically indefinite, but most important, because, as the albuminoid substances are capable of a pathological diffusibility, they are incapable of the nutritive changes to which they were physiologically destined. By these considerations the author concludes that the organism eliminates these substances no longer assimilable, and that the lesion of the kidneys, a depurative organ, does not precede, but is consecutive to this forced elimination. To more confirm his results, he has extended his researches to other organic liquids besides the blood, and when Bright's disease existed he found albumen in the bile, in the sweat and in the saliva, while in other albuminurias the albumen was wanting in these liquids, which demonstrates that, except in Bright's disease, the albumen is due to a particular local state of the renal circulation. If a hypersecretion of the sweat and of the saliva is produced artificially in a patient with Bright's disease, the albumen in the urine is diminished in accordance with its elimination by these secretions, but the quantity eliminated in twenty-four hours remains the same. The consecutive nephritis, which is the most notable anatomical character of the disease, is due to complexed causes that Prof. Semmola has already shown:

1st. Renal hyperæmia caused by the suppression of the cutaneous circulation.

2nd. Irritant action of this hyperæmia according to the excrementitious products accumulated in the blood and the dysocrasic state produced.

3rd. The continuous functional force that the kidneys must employ to liberate the organism of the albumen which has become non-assimilable and diffusible.

Some of the most eminent German professors, who before combated his theory, to-day are his warm supporters. The author thanks especially Prof. Rogedstein, whose works of comparison confirm his theory.—*Giornale Intern. Scienze Mediche.*

BACRATOSIS, OR A SPECIAL SUBLINGUAL FRIBROID PRODUCTION.

F. DeMarinis describes this affection as a hypersarcotic production, found in the oral cavity of poor and neglected children, virulent and fatal. It has never been reported nor described by any author. By the people it is called *Ranocchia*, but not having any scientific name, the author has given one derived from the Greek, for the resemblance which it has to a small frog. In its external and constitutional characters it is entirely different from Ranula. Bacratosis commences on the anterior surface and inferior extremity of the fræenum of the tongue, as a tumor not larger than a lentil, or as a small pearl-whitish stain ; it is nopainful, giving rise to no mechanical or functional disturbance of any sort. After a month or two, this dirty pearl-whitish appear, ance extends in length, breadth and thickness, is either transparent or not, hard or friable, and spreads beyond the frænum on which it lies. As it increases in size, it becomes white as milk, becomes harder, and breaks and divides in its superficies and in its edges, but it never exceeds the size of a large Lima bean; in such a state it produces the greatest possible general alterations, terminating in death.

Sometimes the primordial macula extends anteriorly, resembling a small cauliflower, or extends laterally, and never exceeds the dimensions of half a centmetre in breadth, one centmeter in length, and two or three millmeters in thickness. The children thus affected emaciate rapidly as the disease increases. The animal thermogenesis is lowered, they become extremely leant pale to a marked degree, and death finally supervenes. Bacratosis attacks children enjoying the best of health, although sometimes it is preceded by stomatitis, aphthæ, gastric and intestinal catarrhs or diphtheria. This disease is infective in its last stages, and then is absolutely mortal. Its infection is the same as that of cancer and of equal virulence. The skin becomes straw colored, as in other malignant diseases, the eyes are imbedded in their sockets, the nose pointed, the voice feeble, respiration is abdominal, there is vomiting, with copious and wasting diarrhœas.

DeMarinis defines this disease in its pathological nature as malignant or cancerous, but of a special type, which renders it different from the carcinomatous evolutions and forms.

The treatment consists in caustics and local antiseptics and astringents—generally tonics, cod liver oil and fresh air.—*Giornale Internazional delle Scienze Mediche.*

SIR T. SPENCER WELLS, BART.

The queen has signified her intention to confer a baronetcy upon one of the most eminent surgeons of the day, the president of the Royal College of Surgeons—namely, Mr. Thomas Spencer Wells, of Upper Grosvenor Street, and of Colder's Hall, Hamstead Heath. This honor is officially recorded as a special acknowledgement of "the distinguished services which he has rendered to the medical profession and to humanity." The newly created baronet is the eldest son of the late Mr. William Wells, of St. Alban's. Hertfordshire, by his marriage with Harriet, daughter of the late Mr. William Wright, of East Sheen, Richmond, Surry. He was born in the year 1818, at St. Alban's, and was educated at Trinity College, Dublin. He gained his first medical experience at the infirmary and school of medicine at Leeds, and subsequently studied in the anatomical school at Dublin and at St. Thomas' Hospital. He was admitted a member of the Royal College of Surgeons in 1841, and in 1844 was elected one of the Honorary Fellows created by the new charter. Having become an assisstant surgeon in the navy he saw some active service, both afloat and ashore, before and during the Crimean war ; and he was sent out in 1854-5, under the auspices of Mr. Sidney Herbert, as chief surgeon at Smyrna, and at Rankei, on the Dardanelles. Returning to England at the close of the Russian war, he devoted himself to the study of that branch of professional practice with which his name is associated —namely, ovari-women. He is not only president of the College of Surgeons—in which capacity he delivered the Hunterian oration last year,—but a Fellow of the Royal Medical and Chirurgical Society, and surgeon to her majesty's household ; and at the third centenary of the University of Leyden he had conferred upon him the almost unique degree of an honorary M.D. He is the author of several important surgical works, especially on those improvements in operative surgery to which he has specially devoted himself. He married, in 1853, Elizabeth, daughter of the late Mr. James Wright, solicitor, of New Inn, London, and of Sydenham, Kent, by whom he has a family.—*Illustrated London News*, April 28, 1883.

EXTIRPATION OF A FIBROMA OF LEFT KIDNEY AND SUPRA RENAL CAPSULE, WEIGHING 37¼ POUNDS—RECOVERY.

Dr. Reinhold Buntzel reports (*Berl. Klin. Woch.*) a case of a young woman, 33 years old, who had noticed five years ago a tumor rapidly increasing in size. It had already reached the size of a man's head, and she sought the advise of Spiegelberg, who after an examination declined to operate on account of the extremely reduced condition of the patient. Later, she consulted gynæcologists and surgeons, who made examinations and explorations with no results, and each one declined to operate. Notwithstanding every sort of treatment, the tumor constantly creased ; intense acute pains were felt in the left foot, radiating up the thigh. Digestive troubles came on, and the patient rapidly emaciated. The tumor now extended from the xiphoid appendix down to the superior border of the symphysis pubis, filling all the abdomen. The uterus was free and mobile. No ædema nor ageites. The urine was never albiminous. An operation was made at the request of the patient with all antiseptic precautions á la Lister. The abdominal incision extended from the xiphoid appendix to the superior border of the symphysis pubis. The tumor when laid bare presented some signs of fluctuation, and in an effort to diminish its size, was aspirated, but with no result. The hand could not be introduced behind nor below the tumor to ascertain its relations. It was detached partly with the hand and partly with the bistouri, some parts being adherent to the peritoneum, and arteries of considerable size were tied.

The growth when extirpated weighed 37¼ pounds, and was a fibroma of gigantic dimensions, which had originated in the suprarenal capsule.

The operation lasted two hours and a half. Chloroform was administered, and it was borne well until after the extirpation of the tumor, when symptoms supervened, which necessitated the suspension of the anæsthetic, a resort to artificial respiration, and some hypodermic injections of ether, etc. In four weeks the patient left the bed, and made a rapid recovery.—*Archives Générale de Médicine.*

LEAD PALSY CAUSED BY THE USE OF A HAIR-DYE.

M. Angier, professor of pathological anatomy at Lille, has had occasion to observe a case of lead poisoning, remarkable for the variety of the cause and the difficulty of its diagnosis. It was that of a female sixty-two years of age; feeble; subject to an obsitnate constipation, without colic, who was gradually taken for about a year with a feebleness of the superior limbs, from the forearms to the shoulders. She was treated with several remedies without any relief, and without any knowledge of the true cause of the paralysis. When M. Angier saw the patient; the attitude of the superior members, the "wrist drop," and the complete impotency of the extensor muscles, he immediately thought of lead palsy, and actually discovered a well-marked blue line along the edge of the gums, but there was nothing in the aliment-ation, in the habitation, nor in the profession of the patient, that could suggest the least suspicion as to the way in which the lead had been absorbed. At last it was discovered that the patient, for over five years, had been used to dye her hair, naturally white, with a black cosmetic, which, after an analysis by M. Schmith, was found to contain some sulphate, some iodide, and some chromate of lead. This evidently was the cause of the poisoning. The use of the dye was suspended, and after a month of treatment with sulphur baths, with iodide of potassium, and with faradization, the superior limbs regained their movements, and the general state of health was re-established.—*g. Méd. de Lille, et Par. Méd.*

PARENCHYMATOUS INJECTIONS OF HYPEROSMIC ACID IN TUMORS.

There was a case in the surgical clinic of Prof. Winiwarter, at Liege, of a man affected with a sarcoma of the size of the head of a child, situated in the right lateral region of the neck. On account of its intimate relation and adhesion to the great vessels and nerves of the neck, it was impossible to resort to extirpation. Dr Winiwarter then thought to try the effect of parenchymatous injections of hyperosmic acid (a substance used for histological purposes). For fourteen days he made a daily injection of three drops of an aqueous solution of a hundredth part of hyperosmic acid, with a Pravaz syringe. After this time, the tumor had entirely softened, and the mortified substance, changed into a serous pus, was eliminated through openings made in the skin. These incisions rapidly cicatrized, the infiltration diminished day by day, until after a month the tumor entirely disappeared. The integument that covered it remained intact. There was not the least sign of heat or inflammation, and the general state was not modified in any manner. After this, the injections of hyperosmic acid were employed with success in a sarcoma situated in the region of the shoulder (a relapse after a disarticulation of the humerus); also in a case of multiple lymphomas of the neck, and in scrofulous adenitis of the neck, and in other tumors of this sort.

In cases of carcinoma of the glands, the result was null, although a half syringeful of Pravaz of a 1 per cent. solution had been injected.

The principal advantage of hyperosmic acid seems to reside in the property of not acting on the healthy tissues, and in its having a limited action at the point of injection. Many cases of lymphoma, which before were treated for months with every possible means, with no results, disappeared under the influence of the injections of hyperosmic acid.—*Revue Médicale.*

MICROBES IN MARINE FISHES.

Ollivier and Richet have discovered, by a microscopical examination, microbes in living fishes, killed immediately after being caught; in the sea-eel, the turbot, the sea dog, the shark, the whiting, the red mullet, and in several others. Only in one fish (the sea dog) no microbes could be discovered. In one sea eel, numerous microbes were discovered in the lymphatic fluid, but none in the blood. Except in these two cases, all the parts of the organism of the fishes examined presented large quantities of bacteria. These parasites were especially numerous in the peritoneal fluid, in such amount as to render impossible the counting of them. They are less abundant in the blood than in the lymph, sometimes requiring careful attention to discern them. Most often these bacteria are bacilli, long or short, generally slender, and terminating in a spindle extremity, animated with little oscillatory movements. They are colored by the picro-carminate of ammonia and by the aniline colors. Some presented spores in the middle or at the extremity of the rods. Sometimes the spores, becoming free, were seen swimming at the side of the rods. The authors have made, with the microbes, culture experiments and experiments of occlusion; the latter have been made with parraffine melted at 140 degrees.—*Archives générale de Médicine.*

PHENIC ACID.

Van Hoye has written a thesis with a great conviction acquired through numerous series of experiments, some of them ersonal. Here the principal conclusions—doses of phenic acid with no action on the normal temperature, are sufficient to lower a febrile one, and the lowering is effected in all febricitants, as much in single phlegmasia as in the infective pyrexia. The decrease of temperature commences immediately after the absorption of the remedy ; it oscillates from 1 to 3 degrees centigr. When the antipyretic action of the preceding doses is exhausted, a rigor and all the phenomena of a febrile attack comes on, in the same time the temperature arises to the primitive degree, and probably higher. When a new dose is administered in time, it may stop the attack, or prevent it. Sufficient doses to produce the full antipyretic effect, have no immediate effects on the feverish. In the beginning of the fever fifty centigr. given by the rectum are sufficient in all cases. In general the dose can be progressively increased to 2 grams at a time, and 12 grams a day. One gram given at once has been sufficient in some individuals, of a special susceptibility, to produce a thermic depression, even down to 34 5°, centigr. Such an exaggerated lowering has had no ill effects in any of the febricitants. Pulmonary congestions are to be feared and avoided. The antipyretic properties of phenic acid are to be reserved to combat the hyperthermа of the continued and intermittent fevers.—*Rundschau.*

CHLORATE OF POTASSIUM POISONING.

Four children died from the toxic effects of chlorate of potassium. The solution consisted of chlorate of potassium 7½ grains, tilia infusion 175 grains, with little syrup of orange flowers, taken at tablespoonful doses ten to fifteen minutes apart. Bronardel and L'Hote, after a thorough judicial investigation, have reported in the *Annales de Chimie* the following conclusions : That chlorate of potassium, taken in relatively small but frequent doses, may produce such toxic effects as to produce death. That the chemical researches to find the chlorate of potassium in the organisms must be towards the finding the salt itself, which normally does not exist in the body : to find the chloride and the potassium separately, normal constituents of the organism, would be insignificant. The symptoms of poisoning are divided as following :

(*a*) Cutaneous phenomena, known by the appearance first of bluish stains, then cyanotic, and sometimes icteric.

(*b*) Gastro-intestinal derangements, produced by diarrhœa and greenish vomiting.

(*c*) Derangements of the urinary secretions; if the quantity of the salt taken is small, the urine increases; but if in such quantity to produce toxic spmptoms, at first is diminished or arrested, and then becomes abundant, bloody and albuminous.

(*d*) Derangements of the nervous system, shown by collapse and coma terminating in death.

AFFECTIONS OF MENSTRUATION IN THE TYPHOID CONDITION.

Brierre de Boismont, Vogel, Gubler, Raciborshy, etc., have already studied them, but the results have been contradictory. Barthel proves, in the first place, that the menstrual period is never absent when the date of its appearance is about in the fifth day of the invasion of the disease ; menses will more frequently appear when their epoch is from the sixth to the fourteenth day of the invasion ; beyond this time they will be absent without exception. Amenorrhœa is most frequent in the abdominal typhoid. The character of the sanguinous discharge differs according to the state of the health. During the petechial typhus, its quantity is diminished; in relapsing fever, is increased. When, during a typhoid fever, the menstruation has been absent, there will be amenorrhœa for two to three months after. Bartbel has not noticed as frequently as Gubler the pseudo-menstrual hæmorrhages. He has valued the proportion about 3 to 5 per 100 of cases.—*St. Petersburg Medical Weekly.*

TREATMENT OF PTYALISM OF THE INSANE.

A notable fact, demonstrated by numerous observations made by Dr. E. Duiat, is the toleration of atropine by the insane. These are his conclusions: 1st, the neutral sulphate of atropine is certainly very efficacious in the treatment of the ptyalism of the insane; 2d, when it does not completely cure the salivation, it notably diminished this symptom, and if it reappears, never reaches the primative amount; 3d, about three weeks are required to cure the sialorrhœa, and the daily dose of the atopine is 1 to 3 milligrammes; 4th, the duration of the treatment seems to be more related to the antiquity of the sialorrhœa than to its abundance; 5th, the insane tolerate extremely well the sulphate of atropine; 6th, good results have also been obtained in cases complicated with bronchrrhœa and epilepsy. An obstinate and copious case of epiphora was also cured.—*Giornale Internazionale delle Science Médicale.*

RHEUMATIC OPHTHALMIA.

M. Perrin presented to the Académie de Médecine a second series of observations tending to demonstrate the existence of a conjunctivitis, or better, a purulent ophthalmia of a rheumatic nature. It has the general symptoms of a gonorrhœal ophthalmia. It appears suddenly, and progresses rapidly. The diagnosis is grave. Often its results are : ulceration of cornea and distruction of vision. What distinguishes it from gonorrhœal ophthalmia is that it appears in the absence of gonorrhœa and of purulent ophthalmia, but always after an attack of acute articular rheumatism. This observation shows that rheumatic conjunctivitis is influenced by the state of the weather; that it is more frequent in the cool nights of summer; and, that is is more often the starting point of ophthalmia epidemics in prisons, hospitals, sea vessels, etc.—*Revue Médicale.*

CHOLECYSTOTOMY.

Prof. Langenbeck (*Berl. Klin. Woc.*) recently exterpated a gall bladder from a chronic calculous affection of that organ. He made an incision on the external border of the right rectus muscle, and another at right angles corresponding to the anterior border of the liver. When the abdominal cavity was opened, a ligature was placed around the cystic duct, after which the gall bladder was removed. The case terminated in a good recovery. —*L' Union Médicale du Canada.*

TREATMENT OF CARIES.

Kollman has had occasion to cure four cases of caries by using frictions of black soap only. The obtained results could not be better. The first case was a caries of the sternum and of vertebræ. The usual remedies mostly lauded by classical authors were used internally and externally, but with no success. The author then used inunctions of black soap, already recommended by Dr. Kapper. After a week the patient left the bed and began working. Cod liver oil was also given internally. In other three cases the success of cure was equally rapid. The inunctions were made with a sponge with 15 grams of black soap with a little water, repeated twice a week, and lasting 15 to 30 minutes each. The preferred place of application was the dorsum, from the nucha to the popliteal space of each side.—*Wiener Med. Zeitung.*

REPORT OF MARRIAGES, BIRTHS AND DEATHS IN PARIS FOR 1882.

There have been 21,411 marriages, as follows: Between young men and maidens, 17,619; between young men and widows, 1,206; between widowers and maidens, 1,710; between widowers and widows, 904. Divorced, 12. There have been 62,581 births; 31,828 males and 30.753 females. The number of deaths for 1882, was 58,702; of these 17,411 were of children below five years; the report of births was 106.6 to 100 deaths; 10,342 deaths were caused by phthisis pulmonalis; 7579 due to epidemic diseases, as follows: Typhoid fever, 3,352; small-pox, 661; measles, 1,015; scarlatina, 158; diphtheria, 2,390. The number of suicides was 767; 612 males, 155 females. Lastly, the number of still-births was 5,170.—*Le Pratician.*

EFFECTS OF MEDICINES UPON THE SECRETION OF MILK.

M. Stumpf, in *Dutsch Arch. f. Klin. Med.*, reports the following conclusions: The potassic iodide diminishes the secretion of milk; salicylic acid stimulates it. Alcohol, morphine and lead have no marked effect upon it, not more than pilocarpine. Salicylic acid increases the proportion of sugar in the milk; alcohol increases the proportion of fatty matter. Morphine, pilocarpine and lead produce none of these effects; moreover, the iodide of potassium interferes with the secretion of milk. Alcohol is not eliminated with the milk, while the lead and the salicylic acid are so eliminated, in very small proportion.—*Revue Médicale.*

